# *K-RAS* Mutant Gene Found in Pancreatic Juice Activated Chromatin From Peri-ampullary Adenocarcinomas

**DOI:** 10.1177/2516865719828348

**Published:** 2019-02-19

**Authors:** Joseph Reza, Alvin JO Almodovar, Milan Srivastava, Paula P Veldhuis, Swati Patel, Na’im Fanaian, Xiang Zhu, Sally A Litherland, J Pablo Arnoletti

**Affiliations:** 1 General Surgery Residency Program, AdventHealth, Orlando, FL, USA; 2 Translational Research, Cancer Institute, AdventHealth, Orlando, FL, USA; 3 Institute for Surgical Advancement, AdventHealth, Orlando, FL, USA; 4 Center for Diagnostic Pathology, AdventHealth, Orlando, FL, USA; 5 Center for Interventional Endoscopy, AdventHealth, Orlando, FL, USA

**Keywords:** Pancreatic adenocarcinoma, pancreatic juice, chromatin, biomarker, *K-RAS*, *RET*

## Abstract

External pancreatic duct stents inserted after resection of pancreatic head tumors provide unique access to pancreatic juice analysis of genetic and metabolic components that may be associated with peri-ampullary tumor progression. For this pilot study, portal venous blood and pancreatic juice samples were collected from 17 patients who underwent pancreaticoduodenectomy for peri-ampullary tumors. Portal vein circulating tumor cells (CTC) were isolated by high-speed fluorescence-activated cell sorting (FACS) and analyzed by quantitative reverse transcription polymerase chain reaction (RT-PCR) for *K-RAS* exon 12 mutant gene expression (*K-RASmut*). DNA, chromatin, and histone acetylated active chromatin were isolated from pancreatic juice samples by chromatin immunoprecipitation (ChIP) and the presence of *K-RASmut* and other cancer-related gene sequences detected by quantitative polymerase chain reaction (PCR) and ChIP-Seq. Mutated *K-RAS* gene was detectable in activated chromatin in pancreatic juice secreted after surgical resection of pancreatic, ampullary and bile duct carcinomas and directly correlated with the number of CTC found in the portal venous blood (*P* = .0453). ChIP and ChIP-Seq detected acetylated chromatin in peri-ampullary cancer patient juice containing candidate chromatin loci, including *RET* proto-oncogene, not found in similar analysis of pancreatic juice from non-malignant ampullary adenoma. The presence of active tumor cell chromatin in pancreatic juice after surgical removal of the primary tumor suggests that viable cancer cells either remain or re-emerge from the remnant pancreatic duct, providing a potential source for tumor recurrence and cancer relapse. Therefore, epigenetic analysis for active chromatin in pancreatic juice and portal venous blood CTC may be useful for prognostic risk stratification and potential identification of molecular targets in peri-ampullary cancers.

## Introduction

Peri-ampullary cancer is a broad anatomical designation that includes pancreatic head ductal adenocarcinoma (PDAC), distal bile duct cancer (cholangiocarcinoma), ampullary carcinoma, and duodenal cancer. These tumors arise in immediate proximity to the ampulla of Vater and often cause obstructive jaundice as their presenting symptom. Other tumors such as pancreatic neuroendocrine tumors (PNET) and intraductal papillary mucinous neoplasms (IPMN) may also arise in a similar anatomic location within the pancreatic head. In the absence of distant metastasis and depending on regional vascular relationships, patients affected by these cancers may be candidates for surgical resection with curative intent via pancreaticoduodenectomy. However, recurrence and metastatic risk for postsurgical patients remains high even when complete R0 resection is achieved.^1,2^ In more than 80% of patients, pancreatic cancers have a strong propensity for local recurrence and distant metastasis. We and others have described microscopic remnant tumor cells and circulating tumor cells (CTC) as potential vectors of tumor recurrence that remain or re-emerge after the primary tumor is removed.^3,4^

Preoperative chemotherapy and radiation treatments have gained acceptance for their potential to shrink invasive tumors and maximize chances of complete surgical removal, particularly for borderline resectable and locally advanced PDAC.^1,5^ However, following tumor resection, CTC remain concentrated and active in the portal venous blood^3,6^ providing a reservoir of tumor cells for relapse and metastasis. These CTC are often carrying exon 12 mutated *K-RAS* gene mutations (*K-RASmut*) that provide essential metabolic activation that promotes tumor cell survival and progression. Multiple studies have indicated that tracking of *K-RASmut* gene and gene expression may be a useful tool for monitoring patients for recurrence potential after diagnosis and through treatment.^6–11^

Eshlerman et al^7^ have shown that *K-RASmut* DNA is detectable in pancreatic juice secretions collected during endoscopic examination of persons at risk for PDAC and the level of this biomarker can be correlated with progression to malignancy in these patients.

Following pancreaticoduodenectomy, surgical reconstruction of the gastrointestinal (GI) tract requires the pancreatic remnant to be anastomosed directly to the small bowel (pancreaticojejunostomy) or the stomach. Placement of a temporary, externally draining, pancreatic duct stent is sometimes used at the time of surgery to prevent pancreatic secretions from leaking and causing pancreatic fistula. This stent also allows for access to pancreatic juice for 1 to 2 weeks post surgery, providing the potential for biological sampling and detection of remnant tumor-derived components and metabolites during the recovery period.^12,13^

Due to the caustic, digestive enzyme-rich nature of pancreatic juice, live pancreatic ductal cells cannot be readily detectable as those isolated from the circulatory system in these patients.^14^ We hypothesized that *K-RASmut* and other candidate tumor gene DNA present in the postsurgical pancreatic juice may be a useful indicator of residual tumor cell presence among patients with peri-ampullary carcinomas undergoing pancreaticoduodenectomy. In addition, potential detection of *K-RASmut* DNA in activated chromatin could be characterized as an indicator of recent tumor cell viability and/or active re-emergence post surgery. To test this hypothesis in a pilot study, we collected both intraoperative portal blood CTC and postoperative pancreatic juice from surgical patients and analyzed these samples for *K-RASmut* DNA and acetylated chromatin as the possible indicators of viable remnant cancer cells within the pancreatic duct and the portal blood circulation after pancreaticoduodenectomy.

## Methods

### Patient participants

A total of 37 patients undergoing pancreaticoduodenectomy were enrolled with written informed consent for participation in this study under Florida Hospital Institutional Review Board approval (protocol no. 592917). Patient volunteers consented to collection of intraoperative blood from the portal vein immediately after pancreaticoduodenectomy and collection of pancreatic juice secretions from surgically placed pancreatic stents during their postoperative recovery. Matched samples of both intraoperative portal blood and postoperative pancreatic juice were available in 17 of the 37 consented patients for inclusion in the analyses of this study (demographics listed in Table 1). The underlying pathologic diagnosis for our patient population consisted of PDAC (5, 3 of whom received preoperative chemotherapy), ampullary adenocarcinoma (4, 1 of whom received preoperative chemotherapy), cholangiocarcinoma,^2^ PNET,^3^ IPMN^1^ and benign ampullary adenoma.^2^ All study procedures conformed to the relevant regulatory standards required for ethical research involving volunteer human patients. Sample experimental analyses were conducted blinded to the subject’s final pathology diagnosis and the results segregated to tumor subtype groups after laboratory data collection.

**Table 1.** Study population demographics.

Patient group	N	Sex	Age in years (if n > 2 median (range))
PDAC^a^	5	3 female2 male	65.3 (44-70)
Ampullary adenocarcinoma^a^	4	1 female3 male	70.8 (67-77)
Cholangiocarcinoma	2	1 female1 male	60, 79
PNET	3	2 female1 male	66.0 (63-72)
IPMN	1	1 female	64
Non-malignant ampullary adenoma	2	2 male	76, 84

IPMN: intraductal papillary mucinous neoplasm; PDAC: pancreatic head ductal adenocarcinoma; PNET: pancreatic neuroendocrine tumor.

a Of the 5 patients, 3 with PDAC and 1 with ampullary adenocarcinoma listed in the table received preoperative chemotherapy.

### Blood collection

Blood samples were collected from the 17 individuals undergoing open pancreaticoduodenectomy for the detailed peri-ampullary pathologies (Table 1). A 10-mL blood sample was obtained by direct intraoperative venipuncture of the portal vein with a 21-gauge needle and 10-mL syringe. The venipuncture site was then over-sewn with 5-0 polypropylene suture. Portal vein blood was drawn following dissection of the porta hepatis and pancreatic head resection in all patients. These blood samples were stored in heparin-coated vacutainer tubes and kept on ice until further processing. Specimens were used for isolation of CTC by high-speed fluorescence-activated cell sorting (FACS) and molecular analyses.

### Pancreatic juice collection

As described, a temporary external trans-anastomotic pancreatic duct stent was placed in all patients undergoing pancreaticoduodenectomy. The pancreatic stent is typically left open for about 5 to 9 days during the in-patient hospital stay and the accumulated exocrine pancreatic ductal secretions are collected, measured, and disposed off as waste as a normal part of the postsurgical care. The stent drained pancreatic juice to a sterile external collection bag from which pancreatic juice was collected for the study during postoperative recovery. Study-associated physicians collected the discarded secretions on 2 different days for 9 of the study patients and once during the in-patient stay of the remaining 8 participants. Up to 50 mL of the fluid was collected at each sampling and transferred to a sterile container containing a proteinase inhibitor cocktail tablet (Roche, Indianapolis, IN). The juice samples were processed at Translational Research Core Laboratory of Florida Hospital Cancer Institute for chromatin immunoprecipitation (ChIP)/polymerase chain reaction (PCR)-ChIP-Seq analyses of *K-RASmut* genomic DNA and activated chromatin.

### High-speed aseptic FACS CTC isolation

Nucleated blood cells (NBCs) were separated from red blood cells on Ficoll-Histopaque gradients (Pharmacia/Life Technologies, Grand Island, NY) by centrifugation. The NBC layer near the top of the gradient was collected and washed with rich medium (RPMI 1640 [Mediatech, Manassas, VA], 10% medium 199 [Gibco, Life Technologies, Grand Island, NY], 10% fetal calf serum [Mediatech], 2% antibiotic-antimycotic mix [Sigma-Aldrich, St Louis, MO]) before being immunologically stained with mouse monoclonal antibody fluorescent conjugates directed against CD45 (BD Biosciences, San Jose, CA), EPCAM (BD Biosciences), CD44 (Beckman Coulter, Miami, FL), CD147 (Millipore, Billerica, MA), and/or cytokeratin 19 (BD Biosciences). High-speed aseptic FACS collection (on a MoFlo XDP FACS instrument [Beckman Coulter]) used the immunologic profile of CD44+, CK19+/CD147+, EPCAM+, CD45– as the isolation sort criteria. Sorted CTC were collected and washed with the same rich medium, then analyzed immediately or cryogenically preserved at 1 × 10^7^ NBCs in 90% fetal calf serum (Mediatech/Cellgro-Corning, Corning, NY) with 10% dimethyl sulfoxide (Sigma-Aldrich) for later analysis.

### DNA and chromatin analyses

ChIP isolation of chromatin complex from pancreatic juice samples was performed using modification-specific antibodies for unmodified and acetylated histone H3 as previously described.^3^ Pancreatic juice samples were brought to pH 7-8 if necessary using 1 M HCl or 1 M NaOH (Sigma-Aldrich) and frozen at −80°C for storage. For analysis, juice samples were thawed and precleared of non-specific nucleic acid binding by incubation with salmon sperm DNA Protein A or Protein G beads (Millipore) for 30 minutes at 25°C. Samples were cleared of beads by centrifugation and then diluted 1 to 1 volumetrically with ChIP extraction buffer (50 mM Tris HCl, pH 8, 10 mM ethylenediaminetetraacetic acid [EDTA], 1% sodium dodecyl sulfate [SDS]; Millipore) with inhibitors. The sample was then divided into 4 parts, diluted with ChIP Dilution Buffer (16.7 mM Tris HCl, pH 8, 1.2 mM EDTA, 1.1% Triton X-100, 0.01% SDS, 167 mM NaCl; Millipore) and incubated with salmon sperm DNA Protein A/G beads alone, beads plus 1 µg of non-specific antibody (IgG from mouse or rabbit serum; Sigma-Aldrich), and beads plus 1 µg anti-human histone 3 antibodies (Millipore), or beads plus anti-acetylated histone 3 antibodies (Millipore) for 12 to 24 hours at 4 C. After incubation, the bead-antibody complexes were precipitated and collected by centrifugation and washed successively with Low Salt (20 mM Tris HCl, pH 8, 2 mM EDTA, 1% Triton X-100, 0.1% SDS, 150 mM NaCl; Millipore), High Salt (20 mM Tris HCl, pH 8, 2 mM EDTA, 1% Triton X-100, 0.1% SDS, 500 mM NaCl; Millipore), LiCl (10 mM Tris HCl, pH 8, 1 mM EDTA, 0.25 M LiCl, 1% IGEPAL, 1% deoxycholic acid; Millipore), and Tris-EDTA (TE; 10 mM Tris HCl, pH 8, 1 mM EDTA; Millipore) buffers before the addition of freshly prepared 0.1 M sodium bicarbonate buffer (Thermo Fisher Scientific, Waltham, MA) with 1% SDS (Sigma-Aldrich). Samples were then incubated for 30 minutes at 25°C to detach antibody-antigen complexes from beads. Beads were removed by centrifugation and supernatants brought to 1 M NaCl and incubated for 4 hours at 65°C to de-crosslink formalin-fixed DNA-containing complexes. Once the ChIP-isolated complexes were de-crosslinked, the isolates were treated with RNase A (Millipore) for 30 minutes at 37°C and Proteinase K for 2 hours at 45°C. An equal volume of 100% ethanol (Sigma-Aldrich) was added and the samples were held for 24 to 48 hours to precipitate DNA in the isolates and original untreated juice. The precipitates were collected by centrifugation and DNA purified from the ChIP isolates and genomic DNA samples using a Qiagen Miniprep DNA Isolation Kit (Qiagen, Valencia, CA). Cellular *KRAS* DNA ChIP isolation in these assay conditions was verified by a test run using juice samples spiked with 1000 to 10 000 FACS-isolated cells from patient portal blood sample or CRMCRL 1420 pancreatic cancer cell line cells (ATCC, Manassas, VA, USA). Pancreatic juice genomic and ChIP-isolated DNA samples were amplified in quantitative PCR using TaqMan primers specific for *K-RASwt, K-RAS mut12exon*, and *GAPDH* (Ambion/Life Technologies, Grand Island, NY and Qiagen).

Results from the ChIP isolate relative quantitative PCR analyses were compared using the estimate of expression amplification in quantitative PCR, expressed as the *R* value: *R* = 2(ΔCt Ig – ΔCt specific Ab), where the difference between non-specific antibody binding (ΔCt Ig) and that of specific antibody (ΔCt specific Ab, eg, anti-histone or anti-acetylated histone) is corrected for non-specific background in each patient’s sample.^15^

In addition, acetylated histone 3 ChIP isolates from 3 representative pancreatic juice samples (1 PDAC, 1 ampullary cancer, 1 benign adenoma) were subjected to ChIP-Seq and bioinformatic analyses to confirm the PCR findings (GENEWIZ, South Plainfield, NJ).

*RET* proto-oncogene, a new candidate gene, was unexpectedly revealed in the ChIP-Seq analysis. For subsequent *RET* DNA quantitative PCR analyses, 10 ng of extracted DNA was loaded and amplified using SYBR Green Reaction Mix (Thermo Fisher Scientific) on a ViiA 7 Real-Time PCR System (Applied Biosystems, Waltham, MA) using the primer sequences for *RET* (5′ACA GGG GAT GCA GTA TCT GG and 3′CCT GGC TCC TCT TCA CGT AG).

### Messenger RNA analysis

Portal blood mononuclear cells (PoBMCs) and FACS-sorted CTC samples for messenger RNA (mRNA) analysis were diluted 1 to 2 volumetrically in RNAlater and stored at 4°C for later Trizol RNA extraction. mRNA samples were analyzed by quantitative reverse transcription polymerase chain reaction (RT-PCR) using TaqMan primer sets (Ambion/Life Technologies and Qiagen) specific for *K-RASwt* (UniGene ID: Hs.505033), *K-RAS mut12exon* (5′ACC TTA TGT GTG ACA TGT TCT AAT ATA GT3′ and 5R′GCA CTC TTG CCT ACG CGA T3R′, with probe FAM 5′CCT GCT GAA AAT GAC TGA ATA TAA ACT TGT GG-MGB for exon 12-12Ala, 12Arg, 12asp, 12Cyc, 12Ser, 12Val, and 13Asp mutations, and mutation 12D blocker 5′CCT ACG CCA CCA GCT3′), and *GAPDH* (UniGene ID: Hs.544577). Results from quantitative RT-PCR analyses of patient blood RNA were compared using ΔΔCt values of the *K-RASmut* gene expression with that of the *GAPDH* control. Sequence of the *K-RASmut* RT-PCR product was confirmed in representative CTC mRNA samples (PDAC and PNET) using NextGen sequencing (Beckman Coulter). *K-RAS* gene mutant status was confirmed by pyrosequencing of representative diagnostic formalin-fixed paraffin-embedded (FFPE) tissue samples (PDAC and PNET) from the study patients’ resected tumors.

### Statistical analysis

Mean, standard deviation, correlation, linear and non-linear regression analyses using Prism 5 (GraphPad Software, Inc., 2015, La Jolla, CA, USA) were used to analyze the molecular biological and cell count data of this pilot study. Dependent on the variability, either a Pearson’s parametric or a Spearman’s non-parametric correlation analysis and linear/non-linear regression analyses were used to compare patient progression-free survival (PFS), portal blood CTC number, portal blood CTC *K-RASmut* gene RNA expression, and quantitative real-time PCR *R* value results for *K-RASmut* gene presence in pancreatic juice free DNA and ChIP isolates. The significance level for all tests was set at <.05 (95% confidence). Bioinformatic analyses of the ChIP-Seq peak isolate DNA biomarkers were performed by GENEWIZ (South Plainfield, NJ, USA).

## Results

*K-RASmut* mRNA was detected in CTC from patients with PDAC, ampullary carcinoma, and IPMN, which is considered a premalignant condition (Table 2). Total genomic DNA containing the *K-RASmut* gene was detectable in pancreatic juice within the first 3 postoperative recovery days in the highest levels in *K-RASmut+* tumor patients (including PDAC, ampullary, and cholangiocarcinoma; Table 2). However, no *K-RASmut* DNA was detected in juice from patients with IPMN or non-malignant adenoma. In contrast to CTC mRNA analyses, genomic *K-RASmut* DNA was detected in the pancreatic juice of 1 of 2 PNET patients (Table 2).

**Table 2.** *K-RASmut* analyses and portal blood CTC characteristics.

Patient group	CTC/million blood cells collected, mean (±SD)	CTC *K-RASmut* mRNA+, ΔΔCt mean (±SD)	Pancreatic juice collected (days post surgery)	Juice *K-RASmut* DNA+, ΔΔCt mean (±SD)
PDAC without preoperative chemotherapy	3847 (±3644)	1.025 (±2.511)	1-4	93.74 (±109.7)
Ampullary adenocarcinoma^a^	18 067 ± 25 291	1.533 ± 3.065	1-4	316.8 ± 562.8
Cholangiocarcinoma	2400 (±2226)	0	1-6	93.7 (±119)
PNET	1071 (±1412)	0	1-5	294.5 (±504.2)
IPMN	4892	1.69	4	0
Ampullary adenoma	1009 (±826)	0	2-8	1

CTC: circulating tumor cell; IPMN: intraductal papillary mucinous neoplasm; PDAC: pancreatic head ductal adenocarcinoma; PNET: pancreatic neuroendocrine tumor.

a Contains 1 patient with unusually high CTC counts (54 789/million blood cells sorted).

**Figure 1.**
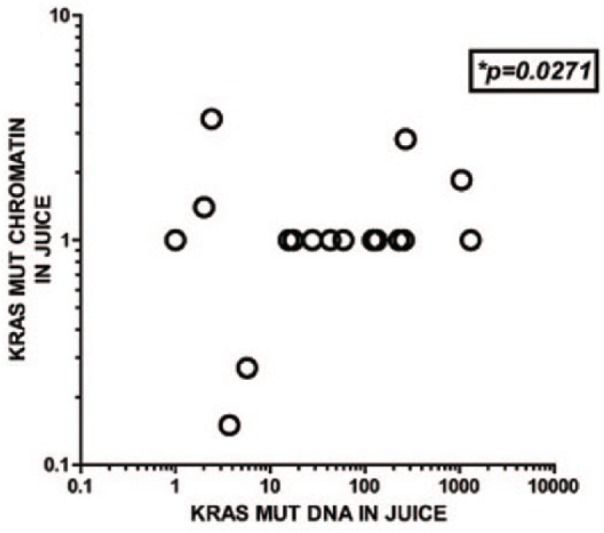
The presence of *K-RASmut* DNA in pancreatic juice correlates with the presence of *K-RASmut*-containing chromatin. Genomic DNA and ChIP-isolated *K-RASmut* DNA found in chromatin and histone acetylated chromatin were extracted from pancreatic juice samples from 17 patients who had undergone surgery for suspected peri-ampullary cancers. The study population included patients that were treated for the conditions listed in Table 2. Genomic DNA detection by quantitative PCR amplification data are depicted as ΔΔCt values of PCR amplification of *K-RASmut* gene RNA expression relative to that of control gene *GAPDH*. Chromatin *K-RASmut* gene locus isolation and amplification are depicted as log *R* values from the relative quantitative PCR analyses. *R* values were calculated as *R* = 2(ΔCt Ig – ΔCt specific Ab).^15^ Non-parametric Spearman’s correlation and linear regression analyses were performed to compare the detection of *K-RASmut* gene in free genomic DNA with that found in chromatin-bound DNA showing a direct correlation between the 2 forms, although this relationship was non-linear (*P* = .0271, Spearman’s non-parametric 1-tailed correlative analysis). Graph represents results from the analysis of 17 patients’ juice samples, with some patients giving samples from multiple days post surgery. ChIP: chromatin immunoprecipitation; PCR: polymerase chain reaction.

**Figure 2.**
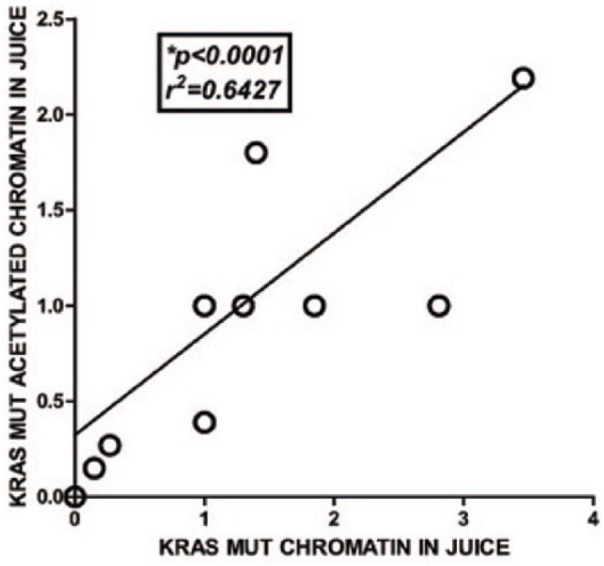
Linear correlation between the presence of *K-RASmut* chromatin in pancreatic juice and detection of acetylated histone on the *K-RASmut* gene locus. ChIP-isolated *K-RASmut* DNA found in chromatin and histone acetylated chromatin was extracted from pancreatic juice samples from 17 patients who had undergone surgery for suspected peri-ampullary cancers. The study population included patients that were treated for the conditions listed in Table 2. The DNA and chromatin *K-RASmut* gene locus isolation and amplification are depicted as log *R* values from the relative quantitative PCR analyses. *R* values were calculated as *R* = 2(ΔCt Ig – ΔCt specific Ab).^15^ Pearson correlation and linear regression analyses comparing the detection of *K-RASmut* gene in chromatin-bound DNA with that found in acetylated histone activated chromatin-bound DNA shows a direct correlation between the 2 chromatin forms, and that this relationship was linear (r^2^ = 0.6427; *P* < .0001, 2-tailed Pearson correlation analysis). ChIP: chromatin immunoprecipitation; PCR: polymerase chain reaction.

ChIP isolation of chromatin containing *K-RASmut* DNA was detectable starting at 2 days post surgery and remained detectable in samples collected up to 6 days post surgery. The detection of chromatin containing *K-RASmut* directly and linearly correlated with the detection of genomic DNA in juice (*P* = .0271; Figure 1). In addition, there was a direct correlation between the presence of *K-RASmut* chromatin and the detection of histone acetylated chromatin containing the *K-RASmut* locus (*P* < .0001; Figure 2).

**Figure 3.**
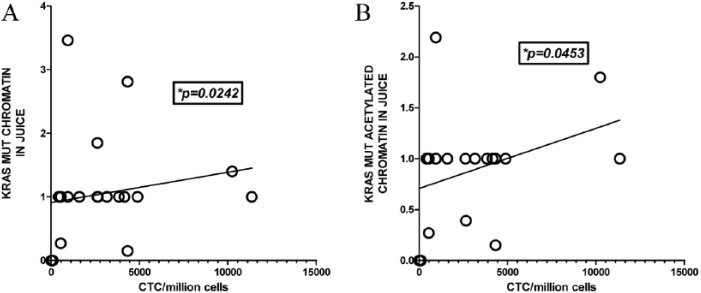
Presence of *K-RASmut* containing chromatin in pancreatic juice correlates with the number of circulating tumor cells in portal venous blood. CTC were isolated from 17 patient blood samples collected intraoperatively from the portal vein during pancreatico-duodenectomy surgery for suspected peri-ampullary cancers. ChIP Isolated *K-RASmut* DNA found in chromatin and histone acetylated chromatin was extracted from pancreatic juice samples collected post-operatively from the same 17 patients who had undergone surgery for suspected peri-ampullary cancers. The study population included patients that were treated for the conditions listed in Table 2. Chromatin *KRASmut* gene locus isolation and amplification are depicted as log R values from the relative quantitative PCR analyses. R values were calculated as R = 2^(DC^_tIg_^-DC^_tspecificAb_^)^.15 Spearman non-parametric correlation analysis done between CTC numbers and *KRASmut* gene chromatin in pancreatic juice samples tested in all 17 patient samples found significance (p = 0.0140 for chromatin; p = 0.0405 for acetylated chromatin). However, one ampullary adenocarcinoma patient sample had unusually high portal blood CTC counts (54,789 million cells sorted). To avoid bias, this sample was left out of the linear correlation analysis. The graphs depict the results of two tailed Spearman non-parametric correlation and linear regression analyses of detection of *K-RASmut* gene in (A) chromatin-bound DNA (p = 0.0242) and in (B) histone acetylated activated chromatin (p = 0.0453) of the remaining 16 patient samples. The analyses indicated linear correlations with number of CTC found in the portal venous blood after pancreatic resection. ChIP: chromatin immunoprecipitation; CTC: circulating tumor cell; PCR: polymerase chain reaction.

PDAC patients with *K-RASmut*+ DNA in their portal blood CTC exhibited *K-RASmut* mRNA expression, indicative of transcriptionally active CTC surviving after primary tumor resection (Table 2). Detection of *K-RASmut* DNA in both chromatin and histone acetylated chromatin in pancreatic juice correlated positively with portal blood CTC numbers (*P* = .0140 and *P* = .0405, respectively). The portal blood sample from 1 patient treated for ampullary adenocarcinoma was unusually high in CTC counts (54 789/million portal blood cells sorted). To test whether this sample was skewing the correlation, we ran the analysis again excluding this sample and found that the correlation with portal blood CTC counts remained significant for both chromatin (*P* = .0242) and histone acetylated chromatin (*P* = .0453) in juice (Figure 3).

Due to the small sample size and limited duration of this pilot trial, no significant correlations were seen in PFS and the laboratory findings of the study.

ChIP-Seq analysis of juice samples from a PDAC, an ampullary adenocarcinoma, and an IPMN patient revealed 3 unique loci found in PDAC: Chromosome 22 (22712914…22713046) which includes the gene locus for immunoglobulin lambda light chain, a gene previously described as upregulated in chronic pancreatitis and pancreatic cancer,^16^ Chromosome 1 (96686856…96687146) encompassing the locus for ribosomal protein L7, and an un-transcribed region on the Y chromosome (11314280…11314344) upstream of *DUX4L17*, the homeobox 4 like 17 locus. Ampullary adenocarcinoma pancreatic juice ChIP-Seq analysis did not yield any unique peak sequences but did indicate an enrichment for Chromosome 4 centrometric locus (51107366…51107480) and a region on Chromosome 7 (143848131…143848736) which includes a currently uncharacterized long non-coding RNA sequence (LOC 105375550). In addition, acetylated chromatin ChIP-Seq analysis of pancreatic juice found a Chromosome 10 (41876818…41877334) genetic locus containing *RET*, a proto-oncogene encoding a tyrosine kinase implicated in medullary thyroid cancer and multiple endocrine neoplasia.^17^ Quantitative PCR analysis of ChIP-isolated chromatin from juice of 17 patients detected *RET* gene loci in 3 of 4 ampullary adenocarcinoma and 1 of 3 neuroendocrine tumors in samples collected at day 3 or later in the postoperative period (Table 3).

**Table 3.** Analysis of *RET* gene locus in pancreatic juice chromatin.

Patient no.	Sample collection (postsurgical days)	Tumor diagnosis	*RET* found in pancreatic juice chromatin (*R* value)
P1	DAY 1 DAY 3	PDAC, T3N1	No No
P2^a^	DAY 1 DAY 3	PDAC, T3N0	No No
P3^a^	DAY 3	PDAC, T1N0	No
P4^a^	DAY 4	PDAC, T3N0	No
P5	DAY 1 DAY 4	PDAC, T3N1	No No
A1^a^	DAY 1 DAY 4	Ampullary adenocarcinoma, intestinal type, T4N1	No Yes; 28.7
A2	DAY 3	Ampullary adenocarcinoma Mixed intestinal and pancreaticobiliary type, T4N1	Yes; 24.2
A3	DAY 2	Ampullary adenocarcinoma, intestinal type, T2N0	No
A4	DAY 1 DAY 3	Ampullary adenocarcinoma, intestinal type, T2N0	No Yes; 2.2
C1	DAY 1 DAY 3	Cholangiocarcinoma; T3N1M0	No No
C2	DAY 2 DAY 6	Cholangiocarcinoma; T3N1	No No
N1	DAY 1 DAY 4	PNET; T3N1	No Yes; 5.0
N2	DAY 1 DAY 5	PNET; T3N1	No No
N3	DAY 1	PNET; T3N1	No
I1	DAY 4	IPMN	No
B1	DAY 2 DAY 8	Ampullary adenoma	No No
B2	DAY 2	Ampullary adenoma	No

IPMN: intraductal papillary mucinous neoplasm; PDAC: pancreatic head ductal adenocarcinoma; PNET: pancreatic neuroendocrine tumor.

a Patient received preoperative chemotherapy.

## Discussion

*K-RASmut* DNA has been detected in endoscopically collected pancreatic juice in patients with IPMN, pancreatic intraepithelial neoplasia, and familial risk for peri-ampullary cancer and may predict future progression toward malignant disease.^7,9^ Presence of free genomic DNA containing the *K-RASmut* gene in endoscopic or early postsurgical pancreatic juice may be the result of residual tumor cell debris from the resected primary tumor or from live tumor cells shedding from the remnant pancreatic duct. Our analysis of *K-RASmut* DNA in pancreatic juice found the gene locus present in activated chromatin 2 to 4 days after surgical removal of the primary tumor. In addition, ChIP-Seq analysis indicated that other unique loci of acetylated, active chromatin are present in PDAC-associated pancreatic juice but was not found in non-malignant adenoma. Further in-depth sequence and expression analyses of more patient samples will be needed to confirm the clinical significance of these sequences. Because the caustic nature of pancreatic juice precludes the collection of live intact cells,^14^ the presence of intact acetylated chromatin in the juice is suggestive of recent presence of live, genetically active cells in the stented duct.

Due to the mixed tumor types, small sample population size and short clinical follow-up, we cannot draw any definitive conclusions as to the predictive value of these biomarkers. Further analysis is warranted to understand the metastatic potential and impact of transcriptionally active *K-RASmut*+ cancer cells remaining in the pancreatic duct and portal venous blood circulation after primary tumor resection. If the detection of *K-RASmut* DNA or other cancer unique activated chromatin loci in pancreatic juice proves predictive of tumor burden or aggressiveness, the analysis of postsurgical pancreatic juice could be a valuable tool for formulating prognostic risk analyses and assessing effectiveness of preoperative systemic therapy as well as completeness of surgical resection.

Correlation of juice *K-RASmut* epigenetically activated chromatin with the number of CTC found post tumor resection suggests there are genetically active tumor cells either re-emerging from the portal circulation or more likely, the pancreatic duct itself. The presence of free genomic DNA early in the postoperative recovery period may be indicative of dead cell debris or of viable cells remaining in the pancreatic duct. However, the decline of detectable, free DNA and the delayed appearance of *K-RASmut*-containing chromatin 2 to 4 days post surgery could suggest *de novo* generation of new viable tumor cells from the pancreatic duct or surrounding tissues. Further investigation into stem cell and mature peri-ampullary tissue biomarkers is needed to deduce the origin and character of the *K-RASmut* bearing cells these chromatin findings represent.

Recent findings of *RET* expression in pancreatic cancer^18^ suggest it as a possible biomarker for perineural invasive cancers, macrophage involvement in cancer survival, and poorer prognosis. The finding of *RET*-containing chromatin in the pancreatic juice of ampullary adenocarcinoma and neuroendocrine patients after 3 to 4 days after surgery suggests further study of its expression as a candidate biomarker for re-emergence of advanced cancers and of importance in designing postsurgical treatment in these aggressive cancers.

## Conclusions

In this pilot study, we have shown that activated chromatin containing *K-RASmut* DNA can be detected in pancreatic juice following the resection of peri-ampullary carcinomas. This may be indicative of residual tumor cell activity that could lead to recurrence as it directly correlated to CTC numbers in the portal venous circulation.
